# A study reaches maturity. 25 years of the HBSC study in Germany

**DOI:** 10.25646/6898

**Published:** 2020-09-16

**Authors:** Matthias Richter

**Affiliations:** Martin Luther University Halle-Wittenberg Medical Faculty, Interdisciplinary Center for Health Sciences, Institute of Medical Sociology

Countless scientific and practical texts have been written about the importance of the health of children and adolescents to both individuals and society. Above all, these texts indicate that childhood and adolescence have long since ceased to symbolise health impairments or a lack of illness. Scientific research in this field aims to detect trends in health developments and to identify target groups for disease prevention and health-promoting measures. Combined with the Robert Koch Institute’s German Health Interview and Examination Survey for Children and Adolescents (KiGGS), the Health Behaviour in School-aged Children (HBSC) study provides the most comprehensive data pool for health reporting and the elaboration and detailed development of preventive measures for this age group in Germany [1]. The differences between the studies in terms of their methodological approach and contextual embedding – the KiGGS study has a national focus and accesses its sample via official residency registries, whereas the HBSC study is embedded at the international level under the patronage of the World Health Organization WHO (with the opportunities for international comparisons that this provides) and accesses data via schools and school classes – mean that the studies complement one another perfectly. For many years, this has led to fruitful, in-depth partnerships ranging from validation studies and joint publications to the establishment of working groups as part of professional scientific organisations.

The German HBSC study is due to mark an important anniversary during the current study cycle: the study will soon be 25 years old and, therefore, is slowly but surely reaching maturity. Since 1982, the HBSC study has been conducted every four years in an increasing number of countries [2]. Germany has participated in the study since 1994 when it was represented by the federal state of North Rhine-Westphalia. However, the 1993/94 cycle was a preliminary study, as Germany had not yet become an official member of the WHO research network [3]. Since then, Germany has participated in seven cycles of the study, which is unique throughout the world and currently covers almost 50 countries. As such, the HBSC study constitutes a key point of reference in international comparative child and adolescent health research. The founders of the study in the early 1980s had truly visionary ideas, and they certainly deserve the greatest respect.

The HBSC study has accompanied a large part of its members’ professional lives, and continues to do so; once you join the study, it is very difficult to let go. Staff turnover has therefore been remarkably low over the years. I can certainly vouch for that; in 1999, I started my first job as a student assistant at Bielefeld School of Public Health under the direction of Professor Klaus Hurrelmann. I was tasked with processing the HBSC data and the results for that year’s international report for the WHO [4]. 21 years later, it is both an honour and a duty to be able to coordinate the study for Germany, with a total of seven study sites.

Sustainability is a key challenge facing any scientific venture. This perhaps applies even more strongly to studies of adolescent health and health behaviour than to studies undertaken in the material or natural sciences, because funding relies more on political trends and the current zeitgeist. In 2004, the main headline from the HBSC study – that young people from Germany were top of the league in terms of smoking – generated a huge amount of interest among media and politics [5]. These days, however, this issue only ever generates marginal interest among politicians and funders because tobacco use has declined significantly in recent years. This, of course, represents a huge success story for disease prevention. However, the fact that this study can still be carried out at all in Germany is particularly impressive. With the exception of a small number of additional samples from the federal-state level, the nationwide HBSC study is self-financed by participating universities and endures solely through the passion and enthusiasm of the researchers involved in the network.

Time and again, the emergence of new health challenges has meant that the health of children and adolescents needs to be placed higher on the scientific, media and political agenda. The numerous and diverse publications and reports derived from the HBSC study, in addition to this issue of the Journal of Health Monitoring, are demonstrative of the high regard in which the HBSC study has been and continues to be held for public health research and practices. Scientific endeavours in this context are aimed at reducing the health impairments faced by children and adolescents, and at promoting their health. Importantly, the HBSC study provides extensive and robust evidence with which to do so and highlights the areas and contexts most in need of action. Moreover, all of this is achieved without external funding and the study results continue to be made freely available… here’s to the next 25 years!
